# Diffuse optical imaging with channel attention fusion network

**DOI:** 10.1117/1.JBO.31.1.016001

**Published:** 2025-12-23

**Authors:** Muhammad Reshail Raza Iftikhar, Ya-Fen Hsu, Min-Chun Pan

**Affiliations:** a National Central University, Department of Mechanical Engineering, Taoyuan City, Taiwan; b Landseed Hospital International, Department of Surgery, Taoyuan City, Taiwan

**Keywords:** diffuse optical imaging, channel attention fusion network, multi-scale feature learning, deep learning, channel attention mechanism

## Abstract

**Significance:**

Traditional optical-property image reconstruction techniques are often constrained by artifacts arising from suboptimal source-detector configurations, the amplification of measurement noise during inversion, and limited depth sensitivity, which particularly impacts the accurate reconstruction of deep-seated anomalies such as tumors.

**Aim:**

To overcome these challenges, this research proposes and implements an end-to-end deep learning framework, Channel Attention Fusion Network (CAFNet).

**Approach:**

CAFNet employs AUTOMAP for domain transformation, feature extraction modules for multi-scale feature learning, and channel attention mechanisms to prioritize critical features. The proposed model is trained and tested on simulated and experimental datasets, utilizing metrics such as mean squared error (MSE), peak signal-to-noise ratio (PSNR), and structural similarity index (SSIM) for evaluating model performance.

**Results:**

CAFNet outperforms traditional and state-of-the-art models, achieving the highest SSIM and PSNR values with the lowest MSE. It effectively reconstructs optical properties with high precision, showcasing its ability to detect and localize inclusions in experimental phantoms. An ablation study is performed to highlight the importance of channel attention in CAFNet.

**Conclusions:**

CAFNet demonstrates a significant advancement in diffuse optical imaging, addressing challenges with noise and domain variability issues. Its robust performance highlights the potential in practical medical imaging applications, offering a reliable solution for reconstructing optical properties in complex scenarios.

## 1 Introduction

In recent years, diffuse optical imaging (DOI) has emerged as a promising functional imaging modality that is noninvasive, cost-effective, and non-ionizing, providing a viable alternative to X-ray mammography and magnetic resonance imaging (MRI) for breast tumor screening and diagnostic applications. DOI has demonstrated significant potential in clinical settings for breast tumor probing.^1^^–^^3^ By employing near-infrared (NIR) light in the 600 to 950 nm spectral wavelength range, DOI enables the measurement of optical property distributions within breast tissue. Key parameters such as the absorption coefficient ($\mu_{a}$) and the reduced scattering coefficient ($\mu_{s}^{\prime }$) are utilized to characterize tissue properties both qualitatively and quantitatively, aiding in the detection of tumors. However, the ill-posed nature of the inverse problem in DOI presents significant challenges for the accurate reconstruction of optical property images.

Conventional DOI relies on iterative methods such as Tikhonov regularization (TR), Levenberg-Marquardt,^4^ and Gauss-Newton optimization,^5^ which minimize the error between measured boundary data and model predictions.^6^ These methods, though effective, are computationally intensive and sensitive to imaging geometry, calibration issues, and detector imperfections.^7^ Consequently, deep learning (DL) has emerged as a promising alternative, building on successes from computed tomography (CT) and MRI, where DL has been used for artifact removal, acceleration, and noise suppression.^8^^–^^11^

U-Net and its derivatives have become standard in image segmentation and reconstruction due to their hierarchical feature extraction and end-to-end learning capabilities.^12^ Although DL is still relatively underexplored in DOI, recent works show growing interest. For instance, attention-based physical U-Net,^13^ multi-scale architectures,^14^ 3D convolutional frameworks,^15^ dual encoders,^16^ and multitask models^17^ have demonstrated potential in reconstructing optical properties from boundary data. In addition,^18^ explores the use of a multi-layer perceptron (MLP) for reconstruction using simulation, phantom, and clinical breast lesion data. Despite this progress, many DL models struggle with the inclusion of low contrast or those positioned near the center, where boundary information is weak and signal attenuation is high.

Some methods attempt to overcome projection interpolation errors by transforming signal-domain data to the image domain via AUTOMAP,^19^ followed by refinement using U-Net-based pixel-to-pixel mappings.^20^^,^^21^ Still, many approaches fail to generalize well across complex imaging scenarios. Although attention mechanisms such as convolutional block attention module (CBAM)^21^ and bottleneck attention module (BAM)^22^ have been widely adopted in CT,^23^ ultrasound^24^ and MRI,^25^ their application in DOI remains limited. Spatial attention has received some attention,^13^^,^^17^ but channel attention and hybrid modules such as CBAM are underutilized in this context.

In this study, we propose the channel attention fusion network (CAFNet), an advanced framework for DOI that integrates AUTOMAP-based domain transformation with a dedicated channel attention module to enhance optical-image reconstruction. By prioritizing relevant feature channels, CAFNet achieves superior reconstruction fidelity compared with AUNet,^24^ a spatial attention-based U-Net, Periodic-Net^14^, and TR as demonstrated through benchmarking for accuracy and computational efficiency. Our evaluation leverages a comprehensive dataset of 10,000 synthetic simulation samples (85% training, 10% validation, 5% test) and 22 experimental phantom cases with known optical properties, designed to mimic realistic conditions. This combined use of simulated and experimental data provides a rigorous assessment framework, validating the capability of CAFNet to accurately reconstruct optical-property images and establishing its potential as a valuable tool for advancing DOI applications.

## 2 Image Reconstruction Schemes

**Fig. 1:**
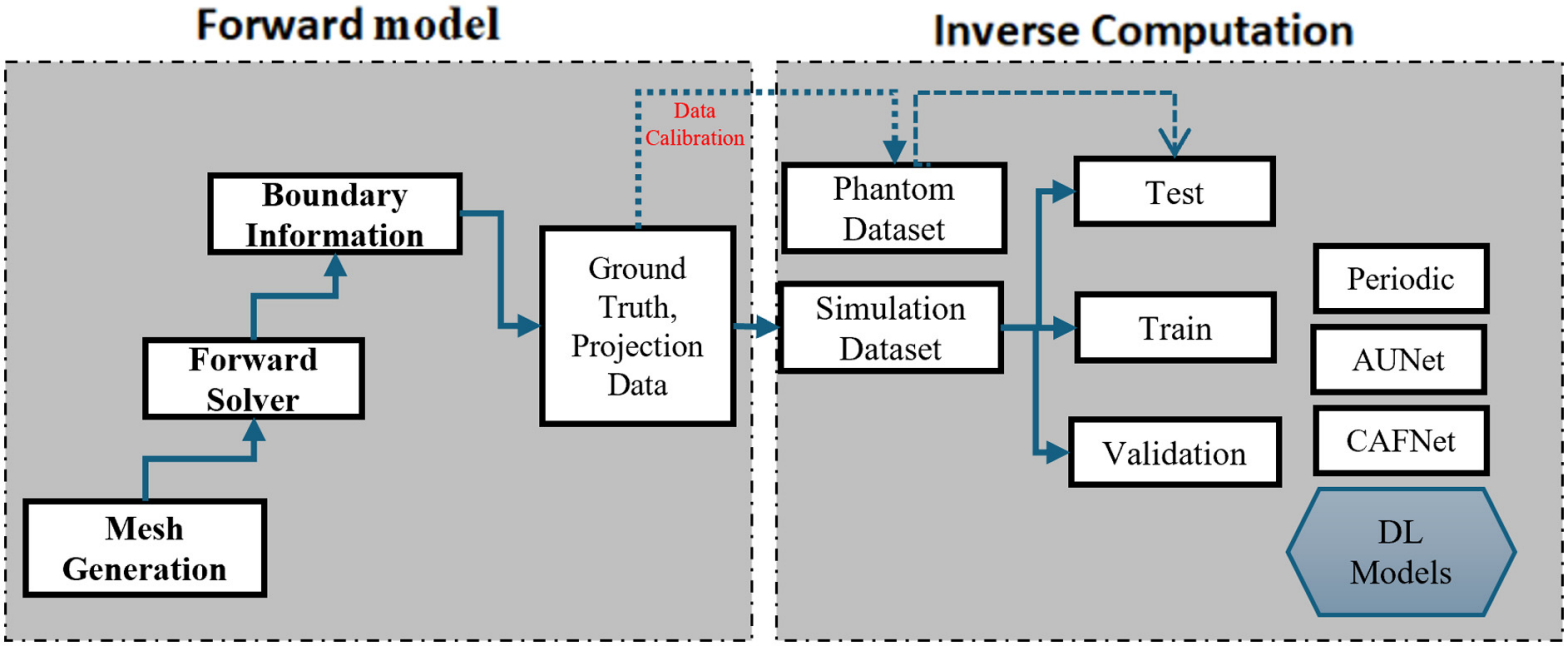
Schematic overview of the proposed optical property reconstruction pipeline.

In this section of the study, optical-property image ($\mu_{a}$ and $\mu_{s}^{\prime }$) reconstruction approaches used are presented: iterative and non-iterative. In the iterative scheme, TR is employed to stabilize the solution of the ill-posed inverse problem. By contrast, the non-iterative approach leverages deep neural networks (DNNs), specifically CAFNet and AUNet, to directly estimate the optical properties from boundary measurements. Figure 1 illustrates the complete schematic of the proposed optical property reconstruction pipeline via DNNs in this study.

### 2.1 Iterative Reconstruction Scheme

**Fig. 2:**
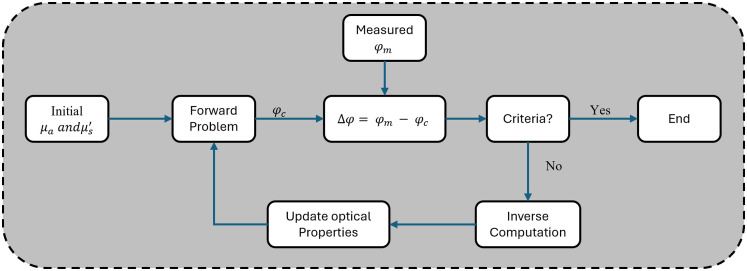
Workflow of iterative reconstruction algorithms.

Traditionally, the reconstruction of optical properties in DOI has been addressed through model-based iterative methods that require a two-step process. The procedure involves a forward solver to compute boundary values from known optical properties by numerically solving the diffusion equation and an inverse model to estimate internal optical properties from boundary measurements by minimizing the objective function. Figure 2 illustrates an iterative framework employed for solving the inverse problem.

To provide a comprehensive understanding of this reconstruction process, the subsequent section presents the mathematical formulation of the forward problem and describes the application of TR to address the inherent ill-posedness of the inverse problem.

#### 2.1.1 Forward solver

In DOI, the propagation of photons in biological tissue is governed by the radiative transport equation (RTE), which models light-tissue interaction by accounting for absorption, scattering, and emission. A NIR light source illuminates the tissue, and detectors placed at the boundary record the transmitted light for further analysis.

The photon radiance L(r,s^,t), defined as the radiative energy at a position vector **r** and time **t** in the direction s^ satisfies the time-dependent RTE^3^

1c∂L(r,s^,t)∂t+s^·∇L(r,s^,t)+μtL(r,s^,t)=∫4πp(s^′,s^)L(r,s^,t)ds^′+S(r,s^,t), (1)

where *c* denotes the speed of light in the medium, $\mu_{t}=\mu_{a}+\mu_{s}$ is the extinction coefficient (sum of absorption $\mu_{a}$ and scattering $\mu_{s}$), p(s^′,s^) the scattering phase function, and S(r,s^,t) the source term. Direct numerical solution of Eq. (1) is computationally expensive for biological tissues, where scattering dominates absorption, i.e., $\mu_{s}\gg \mu_{a}$. Under this assumption, the RTE can be approximated using the diffusion approximation, yielding the frequency-domain (FD) diffusion equation

$$(-\nabla \cdot D\nabla +\mu_{a}-\frac{-i\omega }{c})\Phi (r)=S_{o}(r),$$ (2)

where the diffusion coefficient *D* is defined as $\frac{1}{3(\mu_{a}+\mu_{s}^{\prime })}$, $\mu_{s}^{\prime }$ represents the reduced scattering coefficient, Φ(r) means photon density, and ω denotes the angular frequency.

To solve Eq. (2) numerically, the equation is discretized using the finite element method (FEM), resulting in a linear system

AΦ=b, (3)

where *A* is the global stiffness matrix assembled from the weak form of the diffusion equation, Φ is the vector of nodal fluence values, and *b* is the corresponding source vector. Equation (3) serves as the forward model that relates tissue optical properties to boundary measurements and provides the foundation for solving the inverse problem. Robin-type boundary conditions are applied to the forward model to compute FD measurements for the simulation dataset.^26^^,^^27^

#### 2.1.2 Inverse reconstruction

The TR is used during inverse computation as a regularization technique for optimization and minimizes the objective function^4^ provided as

$$\underset{\Delta x}{\min}\{\|J\Delta x-\Delta \Phi {\|}_{2}^{2}+\lambda^{2}\|L\Delta x{\|}_{2}^{2}\}=\min_{\Delta x}\|\begin{array}{c} J\Delta x-\Delta \Phi \\ \lambda L\Delta x \end{array}{\|}_{2}^{2},$$ (4)

where the Jacobian matrix *J* is defined as $\left[\begin{array}{cc} \frac{\partial \Phi }{\partial \mu_{a}} & \frac{\partial \Phi }{\partial D} \end{array}\right]$ detailing the rate of change in intensity because of optical properties. ΔΦ describes the difference between computed and measured photon density reflecting the residual error, *L* and λ denote the dimensionless regularization matrix that is taken as the identity matrix *I* and the regularization parameter, respectively. The first term on the left of Eq. (4) represents the data fidelity term measuring the residual norm, whereas the second term is a solution norm that is being penalized to stabilize the reconstruction and enforce constraints. The vector Δx represents the optical properties $[\Delta \begin{array}{cc} \mu_{a} & \Delta D \end{array}{]}^{T}$ that are updated iteratively.

### 2.2 Deep Neural Networks

#### 2.2.1 Attention U-Net

**Fig. 3:**
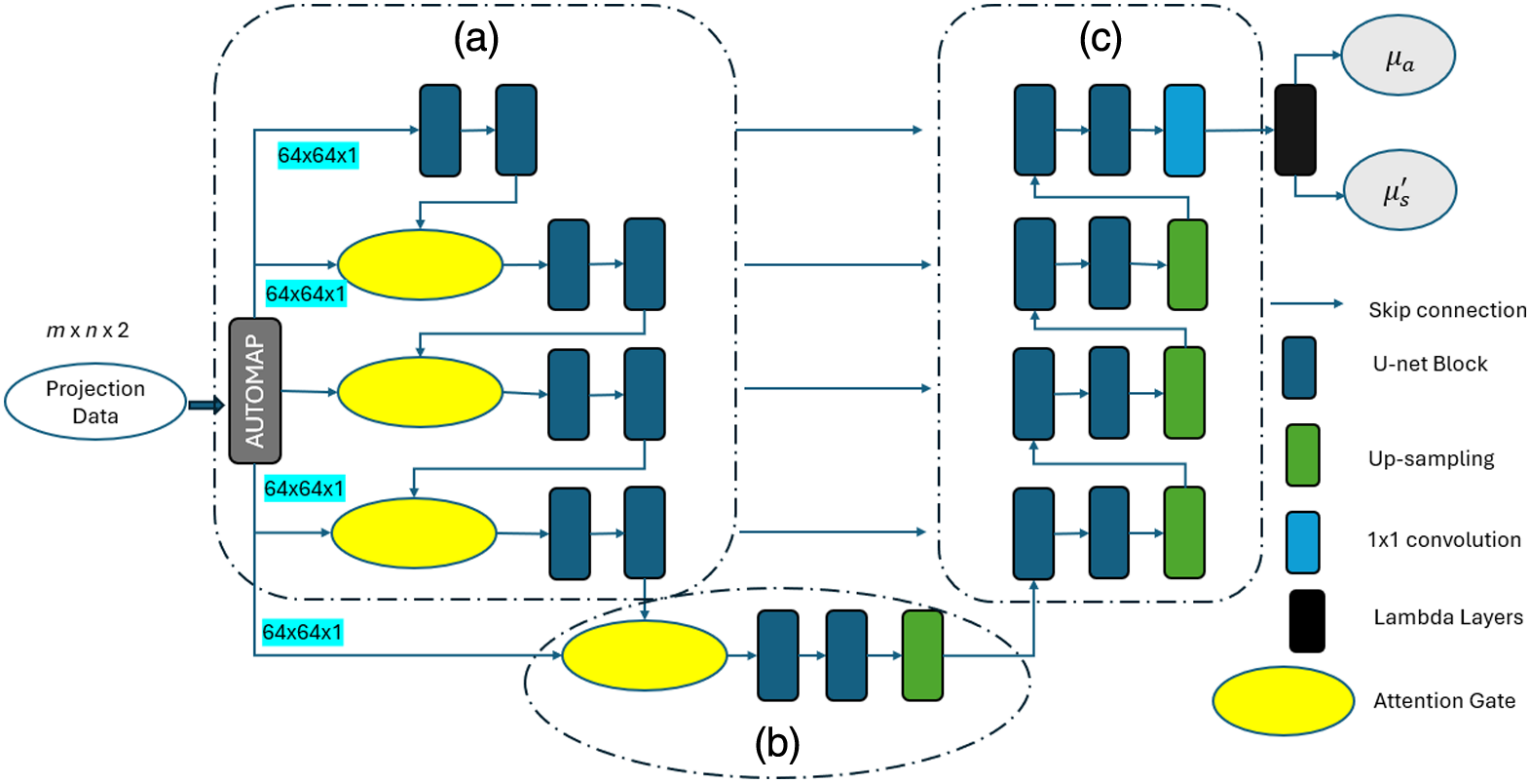
Architecture of the attention U-Net model, comprising (a) an encoder to process the projection data and extract semantics, (b) the bottleneck, and (c) a decoder to upsample the input with lambda layers for reconstructing optical property images.

Figure 3 presents the structure of AUNet^24^ used as a baseline model for optical property reconstruction. The model integrates AUTOMAP^19^ to transform FD boundary measurements (amplitude and phase) into a 64×64-pixel spatial representation. It employs a convolutional encoder–decoder architecture with spatial attention gates in the encoder [Fig. 3(a)] to enhance feature extraction and suppress noise. The bottleneck captures high-level abstractions [Fig. 3(b)], while the decoder progressively upsamples features with skip connections to retain fine spatial details [Fig. 3(c)]. It is ensured that AUNet maintains a consistent 64×64 resolution across all layers, reducing complexity and ensuring precise pixel-to-pixel mapping. A Lambda layer processes the network’s final output, splitting it into two distinct optical property images: $\mu_{a}$ and $\mu_{s}^{\prime }$.

#### 2.2.2 Channel attention fusion network mechanism

**Fig. 4:**
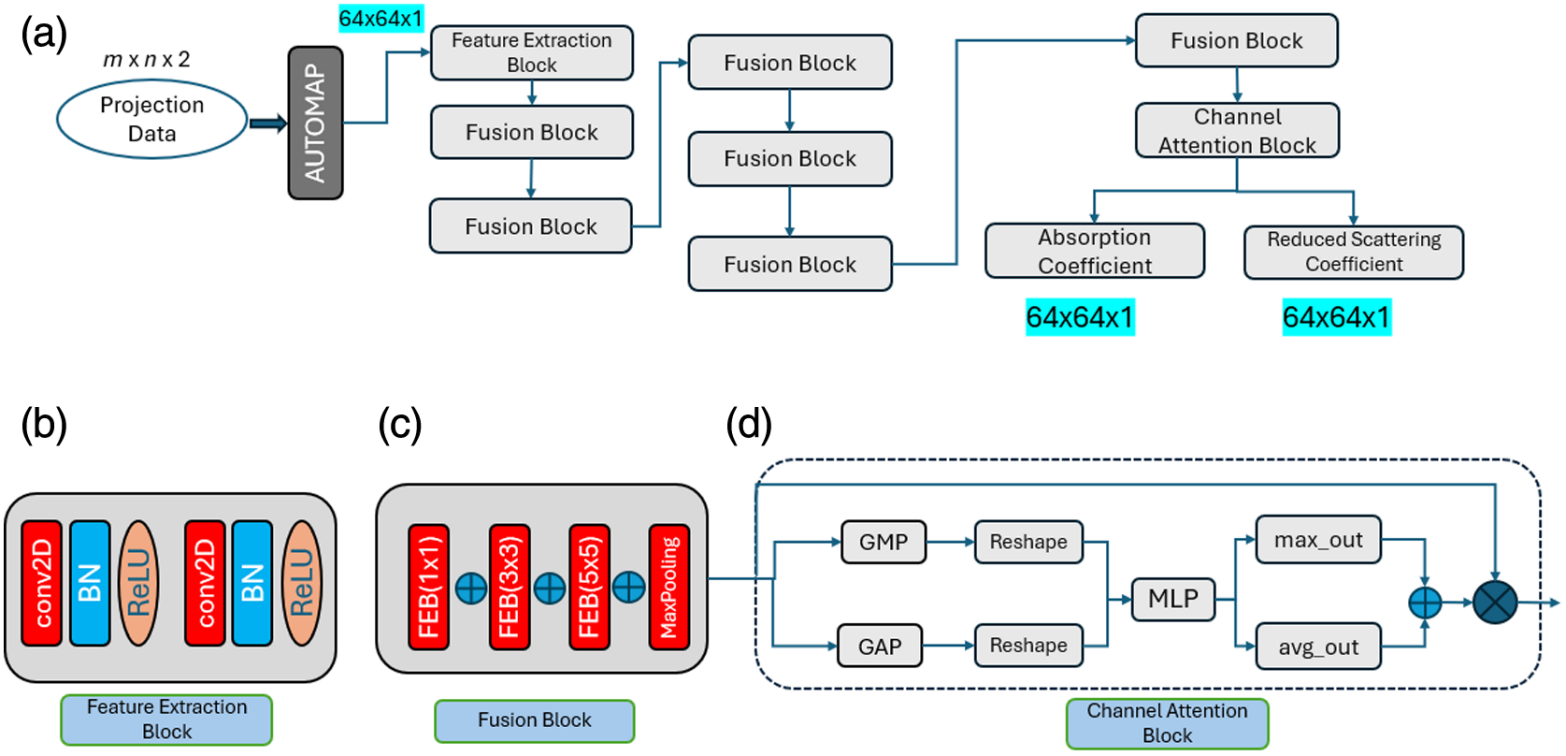
Architecture of (a) CAFNet composed of (b) feature extraction block to extract local features, (c) fusion block to extract global features, and (d) channel attention block to remove noise.

CAFNet distinguishes itself from prior U-Net^14^ approaches by abandoning the conventional encoder–decoder pipeline in favor of a modular design, where channel attention is applied only at the final reconstruction stage. This placement of the channel attention block (CAB) avoids redundant feature expansion–contraction while ensuring that only the relevant channels are emphasized at the output. The CAFNet employs modular architecture, as illustrated in Fig. 4(a), each module is designed to perform a specific task within the network. The input to CAFNet is optical information data with dimensions m×n×2, where 2 represents the channel data (amplitude and phase), and m=16 and m=15 correspond to the projection data. To transform the input from the signal domain to the image domain, the AUTOMAP framework^19^ is utilized. This conversion results in an output with dimensions 64×64×1. The output of the whole network is 64×64×2, where the 2 represents the number of reconstructed optical property images of $\mu_{a}$ and $\mu_{s}^{\prime }$, each with 64×64 dimensions.

The AUTOMAP-generated output is processed by the feature extraction block (FEB), illustrated in Fig. 4(b), which extracts local and low-level features such as edges, textures, and basic shapes. These convolutional layers use 8 filters with a 3×3 kernel size. Each FEB consists of two 2D convolutional layers followed by batch normalization (BN) and ReLU activation to introduce nonlinearity. To enhance feature diversity and capture multi-scale details, the proposed fusion block [Fig. 4(c)] integrates three FEBs with 1×1, 3×3, and 5×5 convolutions, all using the same padding to preserve spatial resolution. This setup enables the network to extract fine-grained details and broader contextual features. After extraction, a MaxPooling2D operation with a 3×3 kernel, a stride of 1, and same padding emphasizes dominant features while suppressing high-frequency noise. The pooled output is refined by a 1×1 convolution with ReLU activation, which compresses the features channel-wise and calibrates them without altering spatial resolution, effectively balancing local detail and global context.

Finally, the outputs of the 1×1, 3×3, and 5×5 convolutions, along with the refined pooled features as illustrated in Fig. 4(c), are concatenated along the channel dimension. This fusion ensures an effective integration of features across different scales, strengthening the network’s ability to detect and represent sparse patterns in the input data.

Figure 4(d) shows the CAB^21^ that is integrated after the fusion blocks in CAFNet to dynamically prioritize channels that carry more relevant information for our reconstruction task, enhancing the network’s ability to focus on meaningful features. This is achieved by emphasizing channels that carry high signal contributions and attenuating those dominated by noise. Feature maps are modeled as $R^{H\times W\times C}$, where each element is defined as

$$X_{i,j,c}=S_{i,j,c}+N_{i,j,c},$$ (5)

with $S_{i,j,c}$ representing the true signal and $N_{i,j,c}∼N(0,\sigma^{2})$ denoting zero-mean Gaussian noise. These feature maps may be spatially corrupted due to noise in the DOI setup.

To estimate channel-wise importance, the CAB employs global average pooling (GAP) and global max pooling (GMP). The GAP operation computes the spatial average for each channel c as

$$z_{\mathrm{avg}}^{c}\approx \frac{1}{H\times W}\sum_{i=1}^{H}\sum_{j=1}^{W}X_{i,j,c},$$ (6)

which effectively reduces the variance of noise to $\sigma^{2}$/(H×W). In parallel, GMP extracts the maximum value $z_{\max}^{c}=\max_{i,j}X_{i,j,c}$, highlighting peak activations indicative of signal-dominant regions. These two summaries are combined additively and passed through a shared MLP, followed by a ReLU and sigmoid activation

$$w_{c}=\sigma (\mathrm{ReLU}(f(z_{\mathrm{avg}}^{c}+z_{\max}^{c}))),w_{c}\in (0,1),$$ (7)

producing soft attention weights $w_{c}$ that scale each channel.

This attention mechanism employs gradual reweighting rather than binary decisions, thus preserving partially informative channels. For noisy channels, low $z_{\mathrm{avg}}^{c}$ and $z_{\max}^{c}$ values lead to a near-zero output from the MLP and ReLU. Mathematically, this would result in $w_{c}=\sigma (0)=0.5$. However, the MLP’s learned weights and biases can effectively push the sigmoid’s input to a strong negative value for truly irrelevant channels, driving $w_{c}$ significantly closer to 0. Conversely, high signal-to-noise ratio channels produce higher $z_{\mathrm{avg}}^{c}$ and $z_{\max}^{c}$, yielding a large positive input to the sigmoid and thus pushing $w_{c}$ closer to 1, amplifying their importance.

The reweighted output feature map is given by $X_{i,j,c}^{\prime }=w_{c}\cdot X_{i,j,c}$, which amplifies signal-rich channels ($w_{c}\approx 1$) and attenuates noise-prone ones ($w_{c}=0$). For noisy channels ($w_{c}\approx 0$), $X_{c}$ will approach 0, whereas for meaningful channels, $X_{c}\approx w_{c}.S_{c}$.

The lambda layers are applied after CAB to produce two outputs that are used to map optical properties, i.e., $\mu_{a}$ and $\mu_{s}^{\prime }$. The final output shape of the optical properties is 64×64×1 each.

## 3 Data Acquisition and Generation

This study leverages (a) numerically simulated phantom data for training and validation and (b) experimental phantom measurements for testing, to evaluate the proposed networks’ ability to reconstruct optical-property ($\mu_{a}$ and $\mu_{s}^{\prime }$) images.

In DOI, computational forward models are critical for simulating photon propagation and generating fluence rate distributions based on given optical properties. In FD systems, these distributions are derived from the amplitude and phase of modulated light signals, providing richer spatial sensitivity to both absorption and scattering. The resulting synthetic measurements form the foundation for training and evaluating inverse models. Following reconstruction, the results are analyzed to assess the accuracy, localization, and reliability of the estimated optical images.

### 3.1 Simulated Phantom Dataset

**Fig. 5:**
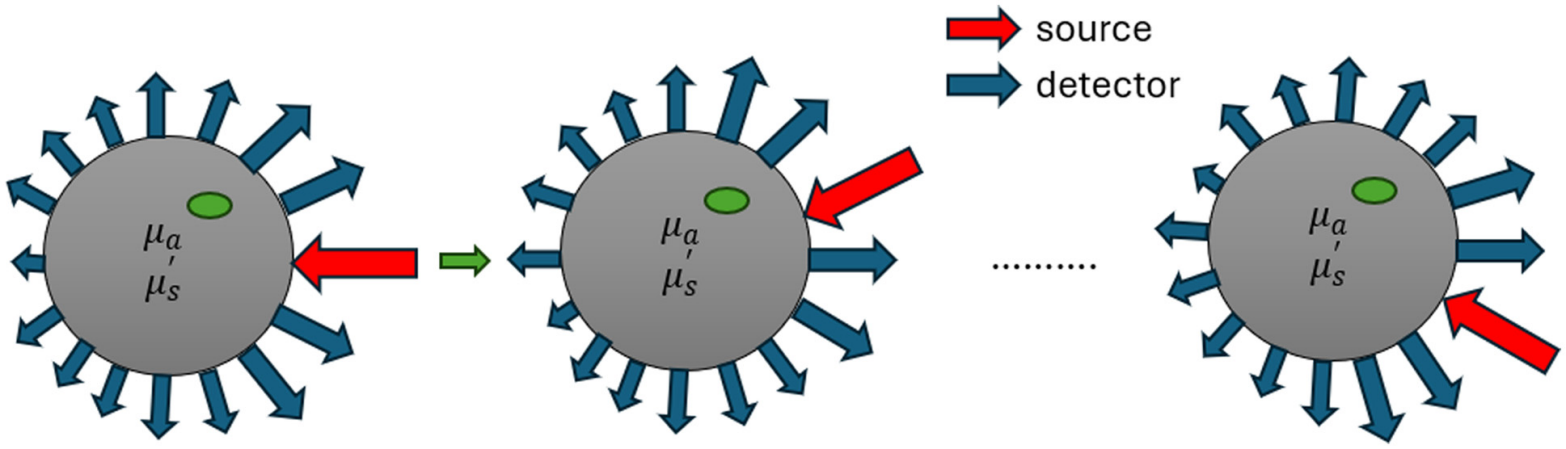
Source-detector positions around circular phantom to generate boundary data.

Image reconstruction in DOI using DNNs requires a large dataset of paired samples (**X**, **Y**), where **X** represents the boundary measurement data (amplitude and phase) derived via forward modeling based on finite element methods and **Y** denotes the corresponding ground truth optical-property images ($\mu_{a}$ and $\mu_{s}^{\prime }$). The boundary measurement data is modeled for human tissue where the reduced scattering coefficient dominates the absorption coefficient ($\mu_{s}^{\prime }\gg \mu_{a}$). In this study, we generate a phantom dataset consisting of 10,000 samples using our in-house simulation framework NIR•FD_PC.^26^^,^^28^^,^^29^ The dataset includes circular phantoms (diameters: 60 to 150 mm), with 1% homogeneous and 99% inhomogeneous cases (44% with one inclusion, 55% with two). Inclusion radii range from 2 to 30 mm, and simulations are performed using frequencies from 10 to 100 MHz. Background optical parameters span $\mu_{a}$ 0.005 to $0.03\;{\mathrm{mm}}^{-1}$ and $\mu_{s}^{\prime }$ 0.5 to $3\;{\mathrm{mm}}^{-1}$. Each sample contains boundary measurements from 16 evenly spaced sources and 15 detectors, yielding 240 amplitude and 240 phase observations (16×15) per sample (Fig. 5). The unstructured finite element mesh and corresponding ground truth images (based on known inclusion parameters) are mapped onto a 64×64×2 grid for reconstruction and evaluation of absorption and scattering coefficients. To mimic real-world conditions, 15% additive noise is applied to the boundary data. Table 1 summarizes dataset parameters used in training (T), validation (V), and testing (Te).

**Table 1:** Simulated phantom dataset properties.

Dataset	Circular data properties
T, V, and Te	8500, 1000, 500
Phantom diameters range	60 to 150 mm
Frequency of operation	10 to 100 MHz
Background $\mu_{a}$ range	0.005 to $0.03\;{\mathrm{mm}}^{-1}$
Background $\mu_{s}^{\prime }$ range	0.5 to $3\;{\mathrm{mm}}^{-1}$
Inclusion contrast range	1.5 to 8
Inclusion radii range	2 to 30 mm
Partition of samples	Homogeneous: 100
	1 inclusion: 4400
	2 inclusions: 5500
Source/detector	16/15

### 3.2 Experimental Phantom Dataset

The experimental dataset^28^^,^^30^^–^^32^ utilized in this study consists of 22 tissue-mimicking phantom samples with circular geometries, each with 50 and 80 mm diameters through the phantom test at the laboratory. The phantoms were fabricated using a silicone matrix doped with carbon and TiO_2_ powders to control absorption and scattering properties, respectively. A single light source and a single detector were employed. The source was sequentially placed at 16 equidistant positions along the phantom’s boundary, and for each source position, the photo multiplier tubes (PMT) as detectors were repositioned to 15 non-overlapping locations, yielding 240 distinct source-detector pairs per sample. As each pair provides both amplitude and phase measurements in the FD, a total of 480 scalar values were collected per phantom. Table 2 presents a summary of the experimental phantom dataset used for testing, and the Appendix lists all individual samples.

**Table 2:** Experimental phantom dataset properties.

Dataset	Circular data properties
Phantom diameters range	50, 80 mm
Freq. of operation	20, 50, and 70 MHz
Background $\mu_{a}$ range	0.006 to $0.01\;{\mathrm{mm}}^{-1}$
Background $\mu_{s}^{\prime }$ range	0.3 to $1\;{\mathrm{mm}}^{-1}$
Inclusion contrast range	2 to 4
Inclusion radii range	5, 10 mm
Samples partition	1 inclusion: 14
	2 inclusions: 8

**Fig. 6:**
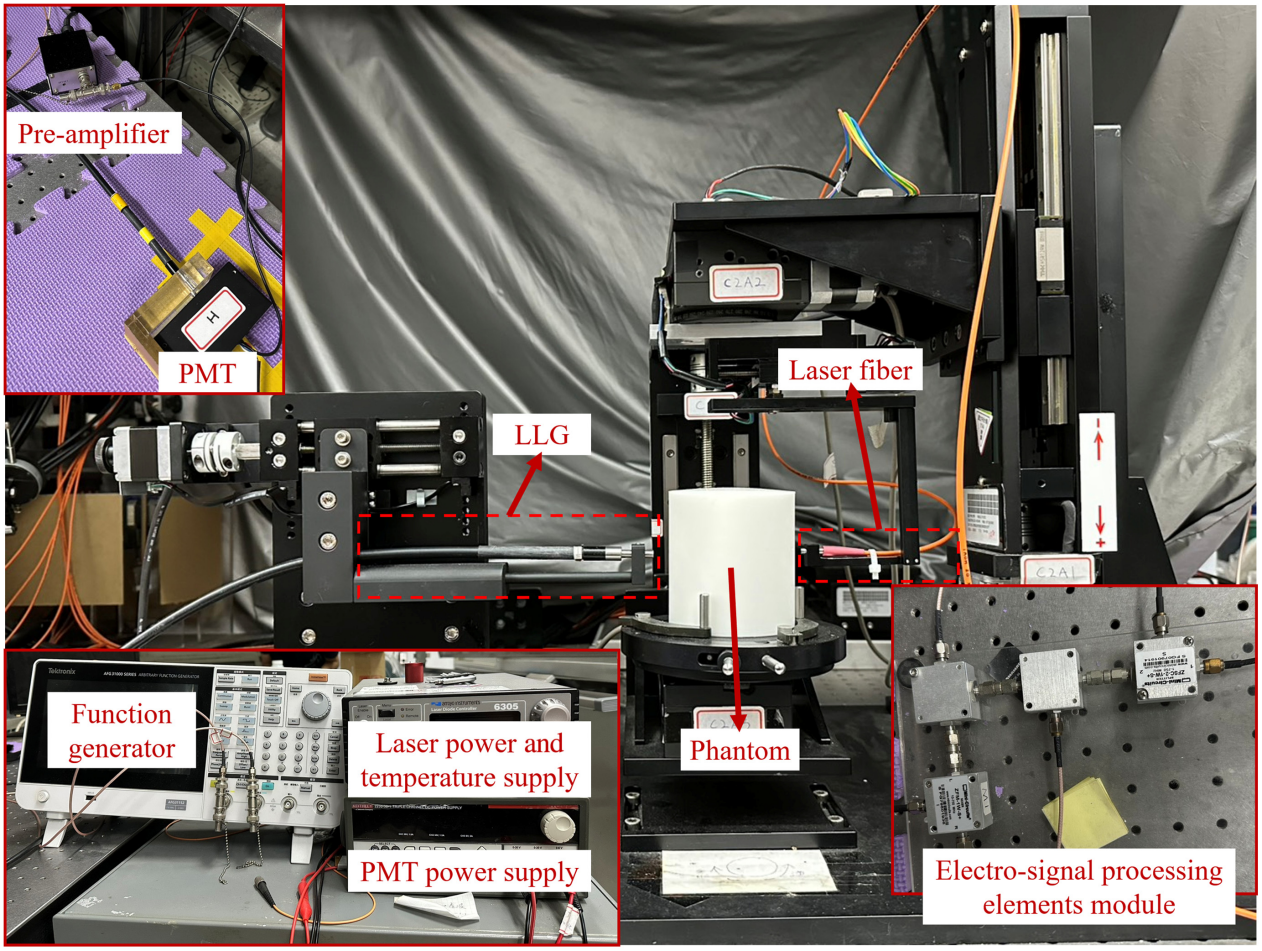
Experimental setup for phantom dataset acquisition, featuring key components including a phantom, laser fiber, and signal processing module.^32^

Due to the turbid nature of biological tissue, photon propagation is highly scattering, resulting in complex trajectories and weak detector signals. Therefore, a calibration procedure was conducted using both homogeneous and inhomogeneous phantom measurements acquired under identical conditions. The resulting calibrated dataset consists of normalized logarithmic amplitude and normalized phase measurements, arranged as 16×15×2 arrays for each sample. These data served as inputs to the deep learning reconstruction model, which outputs 64×64-pixel maps of absorption and scattering coefficients. Figure 6 shows the setup for generating the experimental phantom dataset, including a pre-amplifier, PMT, liquid light guides (LLG), laser fiber, phantom, function generator, PMT power supply, laser power and temperature supply, and an electro-signal processing element module.

## 4 Results

This section presents the results of the proposed DNN-based reconstruction methods evaluated on both simulated and experimental phantom cases. The performance of the proposed models is compared to the traditional TR method and Periodic-Net.^14^ Qualitative assessments, such as image clarity and artifact suppression, are discussed in Sec. 4.1, whereas quantitative metrics including mean squared error (MSE), peak signal-to-noise ratio (PSNR), and structural similarity index (SSIM) results are reported in Sec. 4.2. Additional resolution analyses, such as contrast resolution, size resolution, and contrast-and-size detail (CSD), are presented in Sec. 4.3. An ablation study on a representative case is conducted in Sec. 4.4 to evaluate the contribution of channel attention in CAFNet. Table 3 details the computation environment setup for DNNs implemented in this study.

**Table 3:** Computation environment specifications.

Specifications	Related information
Loss function	Weighted sum of MSE
Learning rate	0.001
Optimizer	Adam ($\beta_{1}=0.5$)
Batch size	Training: 64. Inference: 1
Epochs	100
Framework	TensorFlow
GPU	GeForce RTX 2080 SUPER
RAM	32 GB

Table 4 compares computational efficiency across methods based on training time, inference time, parameters, and GFLOPs. Except for TR (156 s per reconstruction), all methods achieved inference times less than 1 ms, enabling near real-time reconstruction. CAFNet’s 0.331 GFLOPs demonstrate its high computational efficiency and low processing demand.

**Table 4:** Efficiency comparison between CAFNet with other methods.

	CAFNet	AUNet	Periodic
Inference time (ms)	0.81	0.38	0.35
Training time (s)	32	24	8
GFLOPs	0.331	1.32	0.04
Total layers	152	76	174
Trainable parameters	8,201,385	2,902,297	1,258,308

A 5-fold cross-validation is performed and illustrated in Table 5 to assess CAFNet’s generalization ability by training and testing it on different subsets of the data. It helps reduce bias and variance in performance estimates compared with a single train-test split.

**Table 5:** CAFNet cross-validation results across folds.

	Fold-1	Fold-2	Fold-3	Fold-4	Fold-5
MSE	0.009	0.0082	0.0089	0.0105	0.0084

The MSE obtained was 0.0092±0.0009, where 0.0092 denotes the average MSE and 0.0009 reflects the standard deviation across folds. The low variability indicates that CAFNet maintains consistent performance across folds, highlighting its stability.

### 4.1 Reconstruction of Test Samples

**Fig. 7:**
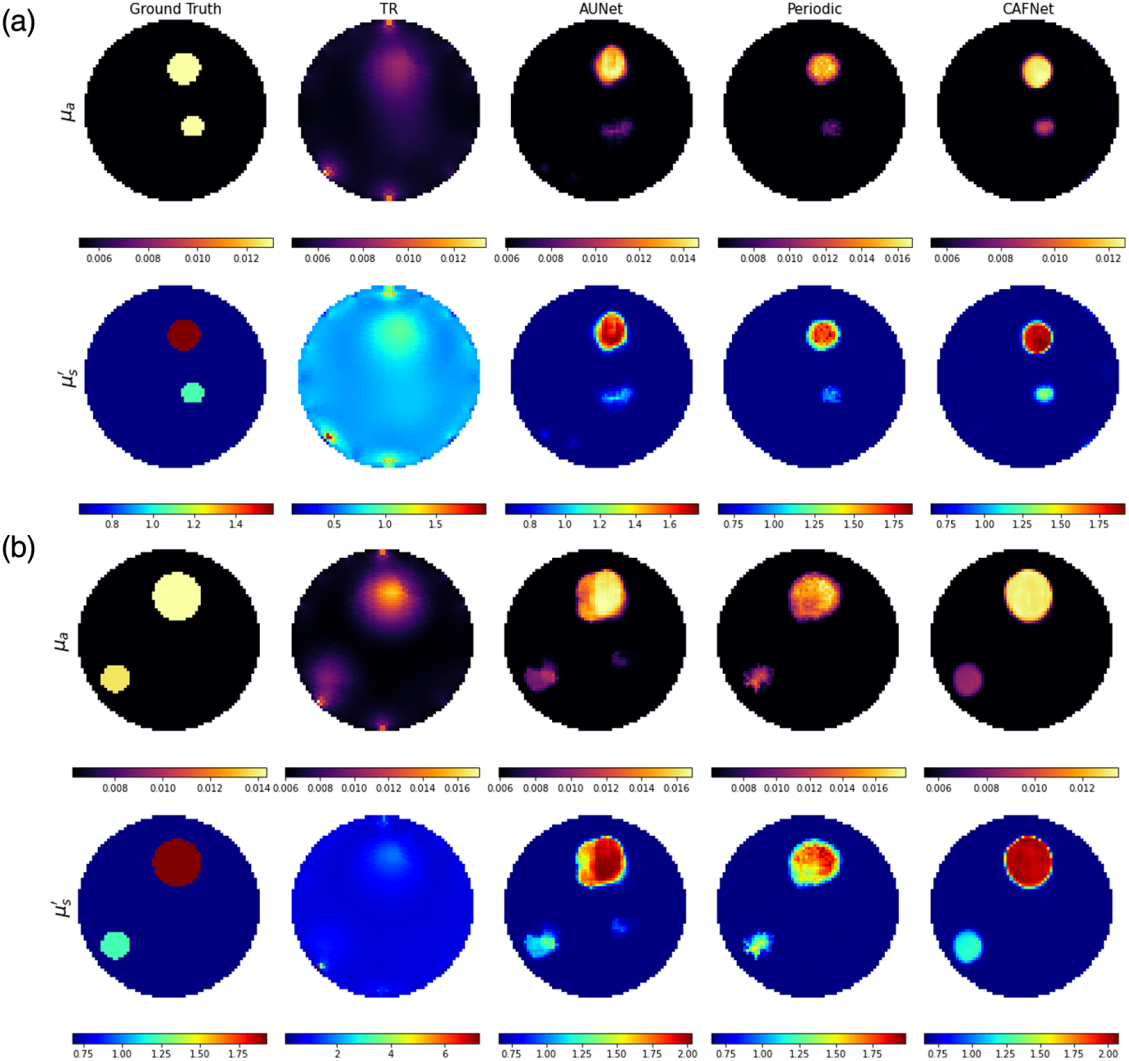
Reconstructed images of optical properties: (a) complex simulated case and (b) simulated test sample for the case referenced in Ref. 14.

**Fig. 8:**
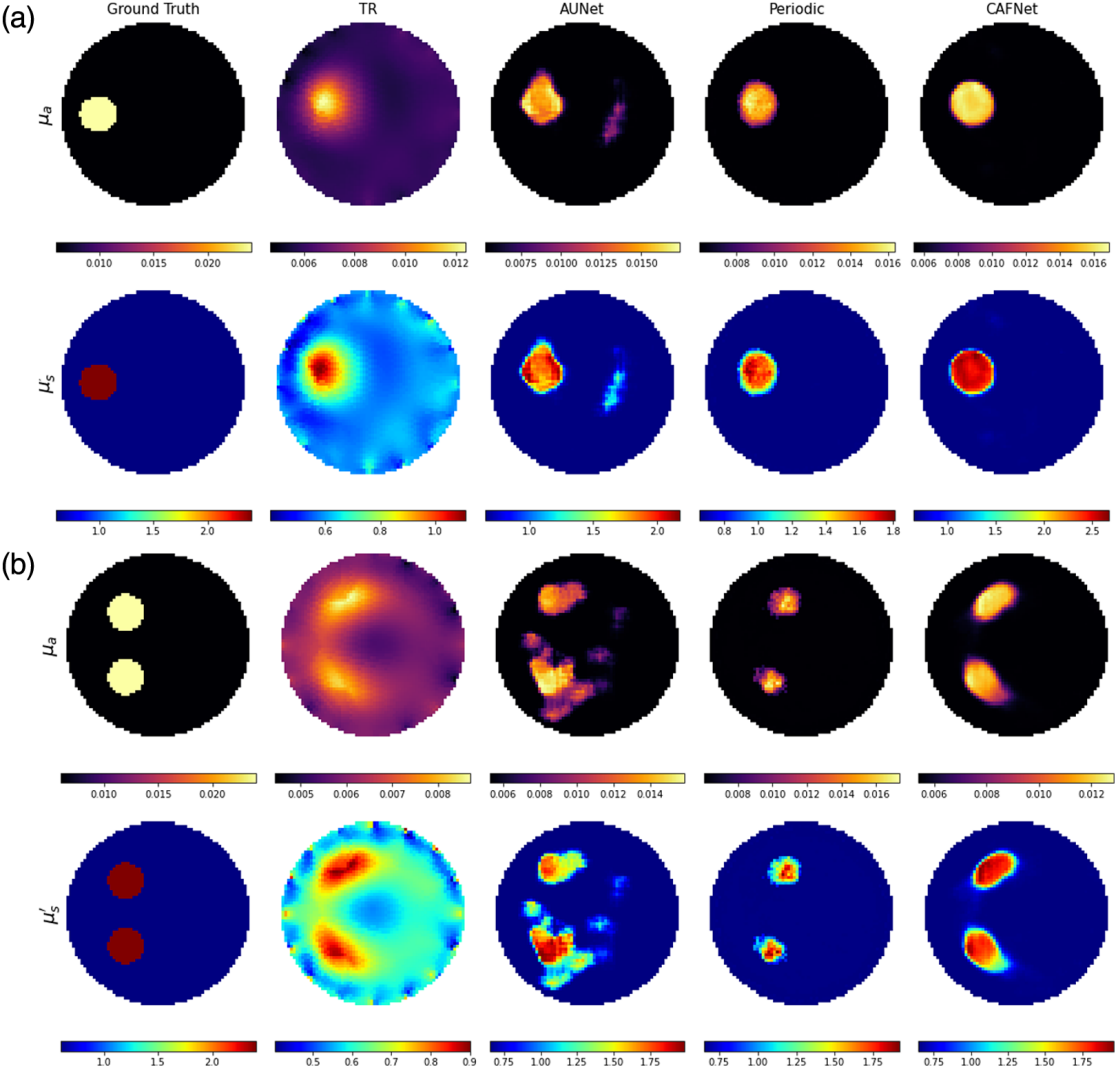
Reconstructed images of optical properties: (a) experimental test sample for the case referenced in Ref. 14 and (b) experimental double inclusion test sample.

Figure 7 presents the reconstructed optical-property images for simulated test samples, whereas Fig. 8 displays the corresponding reconstructions for experimental phantom cases as listed in Table 6. It visually compares each method’s ability to recover spatial features of inclusions under varying complexity. Figures 7 and 8 are organized into two rows representing $\mu_{a}$ and $\mu_{s}^{\prime }$, respectively. The columns correspond to the Ground Truth, TR, AUNet, Periodic-Net, and CAFNet. Notably, the test samples shown in Figs. 7(b) and 8(a) replicate scenarios used in Ref. 14, allowing for objective comparison between the proposed deep learning models and existing state-of-the-art techniques.

**Table 6:** Selected circular simulated and experimental phantom cases from test datasets for verification.

Dataset type	Freq. (MHz)	$\mu_{a}$ (${\mathrm{mm}}^{-1}$)	$\mu_{s}^{\prime }$ (${\mathrm{mm}}^{-1}$)	Diameter (mm)	Radius (mm)
Simulated	10	0.06	0.6	70	11.25, 6.77
	20	0.0051	0.641	70	6.17, 9
Experimental	20	0.006	0.6	50	5.0
	20	0.006	0.6	50	5.0, 5.0

Figure 7(a) introduces a particularly challenging simulated scenario that was purposefully designed to stress-test the reconstruction algorithms. This case includes a centrally positioned inclusion with low optical contrast relative to the background, making detection significantly harder due to minimal perturbation in the diffuse light signal. The central location also causes photons to undergo longer and more scattered paths, resulting in signal attenuation and reduced boundary sensitivity. Under these adverse conditions, TR fails to detect the inclusion, whereas AUNet and Periodic-Net show partial success with notable inaccuracies in shape and contrast. CAFNet, however, successfully reconstructs the inclusion with high fidelity, capturing both geometric structure and intensity, thus confirming its robustness in low-contrast, signal-attenuated scenarios.

In Fig. 7(b), reconstructions are based on simulated data with clearly distinguishable inclusions, serving as a baseline to evaluate general reconstruction performance. The TR method results in heavy blurring and fails to localize inclusions accurately. AUNet improves spatial localization compared to TR but introduces distortions in both inclusion shape and intensity. Periodic-Net yields sharper reconstructions and better feature definition, although residual errors persist in certain regions. CAFNet, by contrast, achieves reconstructions that are closely aligned with the Ground Truth in terms of both inclusion location and intensity, demonstrating its superior accuracy under standard conditions.

In Figs. 8(a) and 8(b), experimental phantom data are used to evaluate real-world applicability. The TR method again shows over-smoothing effects, obscuring important spatial transitions and boundaries. AUNet improves localization but suffers from amplified noise artifacts, affecting image clarity. Periodic-Net produces reconstructions with enhanced sharpness and better-defined features. CAFNet outperforms all other methods by yielding images with higher shape accuracy and superior contrast preservation, closely matching the experimental ground truth.

### 4.2 Statistical Analysis of Reconstructed Images

The reconstruction accuracy of the predicted optical-property images, specifically the absorption coefficient ($\mu_{a}$ and $\mu_{s}^{\prime }$) is quantitatively evaluated using three metrics: MSE,^33^ PSNR,^34^ and SSIM.^35^ Given the ground truth optical-property distribution μ(i) and the corresponding reconstructed values μ^(i), the MSE is computed over N pixels as

MSE=1N∑i=1N(μ(i)−μ^(i))2, (8)

PSNR provides a logarithmic measure of reconstruction fidelity, calculated as

$$\mathrm{PSNR}=10\log_{10}\frac{\max^{2}}{\mathrm{MSE}},$$ (9)

where max is the maximum pixel intensity value. The max value is normalized to 1. In addition, SSIM measures perceptual similarity by considering luminance, contrast, and structural information, defined for images μ and μ^ as

SSIM(μ,μ^)=(2μμμμ^+C1)(2σμμ^+C2)(μμ2+μμ^2+C1)(σμ2+σμ^2+C2), (10)

where $\mu_{\mu }$ and μμ^ denotes local means, $\sigma_{\mu }^{2}$ and σμ^2 are variances, σμμ^ is covariance, and $C_{1}$ and $C_{2}$ are constants to stabilize the calculations. The constants here are set to $C_{1}=0.001^{2}$ and $C_{2}=0.002^{2}$.

**Fig. 9:**
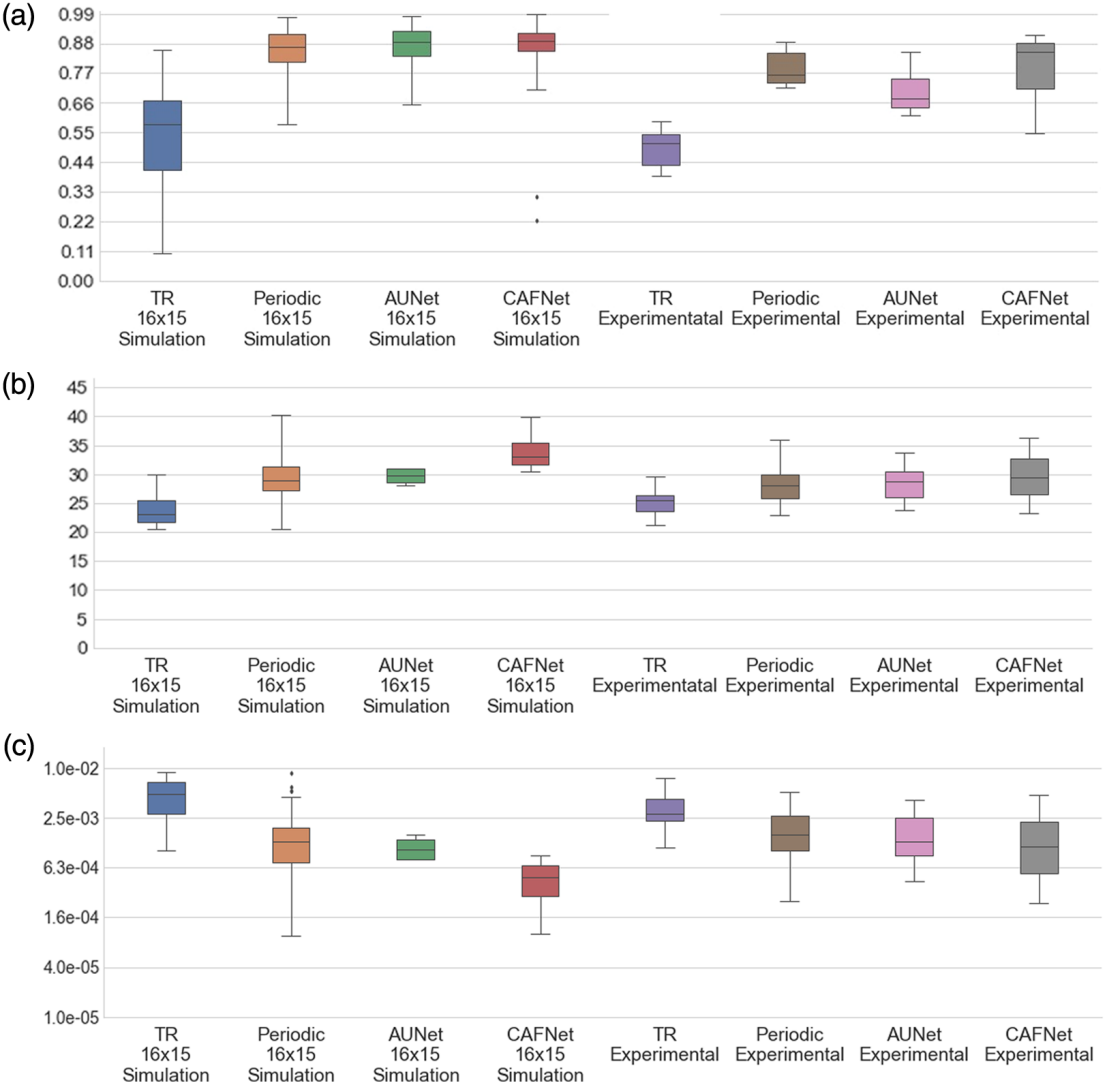
Evaluation of PSNR, SSIM, and MSE for $\mu_{a}$ (a) SSIM, (b) PSNR, and (c) MSE for 500 simulation test data samples as well as 22 experimental data samples.

**Fig. 10:**
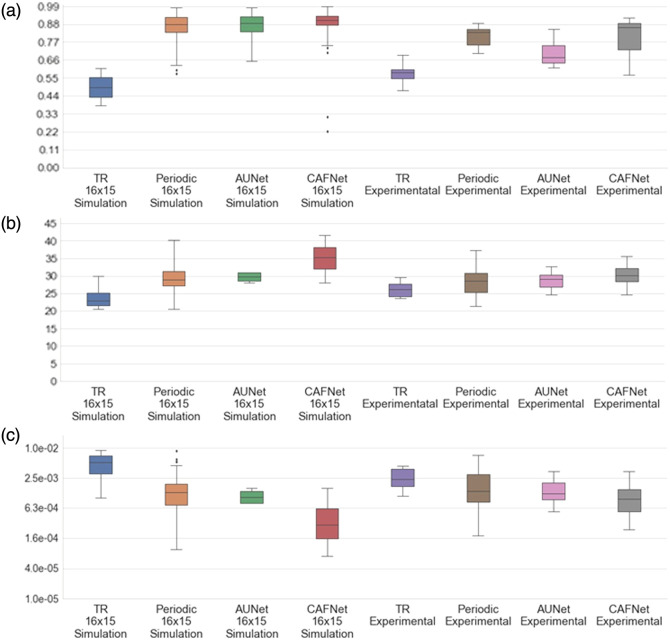
Evaluation of PSNR, SSIM, and MSE for $\mu_{s}^{\prime }$ (a) SSIM, (b) PSNR, and (c) MSE for 500 simulation test data samples as well as 22 experimental data samples.

The SSIM, PSNR, and MSE for absorption coefficient ($\mu_{a}$) is shown in Figs. 9(a)–9(c), respectively, whereas Fig. 10 illustrates the statistical analysis of reduced scattering coefficient ($\mu_{s}^{\prime }$). In both Figs. 9(b) and 10(b), the PSNR analysis indicates consistently superior performance of CAFNet, exhibiting higher median values for both simulation and experimental datasets. This highlights its robustness in preserving image quality under varying conditions. Periodic-net also performs competitively, particularly in the simulation dataset, but its performance shows a marginal decline when applied to experimental data. AUNet and TR, on the other hand, exhibit comparatively lower PSNR values. Although AUNet performs moderately well in simulation, its performance deteriorates significantly in experimental phantom cases cause to domain shift.

For SSIM evaluation in [Figs. 9(a) and 10(a)], which quantifies structural similarity, CAFNet again outperforms other models, effectively reconstructing high-quality structural details in optical images.

The difference between simulation and experimental performance is minimal for CAFNet, demonstrating its strong generalization capability. By contrast, AUNet and TR exhibit relatively lower SSIM values, further reflecting their limitations in maintaining structural integrity across datasets. MSE analysis [Figs. 9(c) and 10(c)] reveals that CAFNet achieves the lowest error rates, followed closely by periodic-net in simulations. TR, however, shows significantly higher MSE values, suggesting inferior reconstruction accuracy. These results collectively confirm the superior performance of CAFNet across all metrics, particularly its robustness and generalization to experimental data, positioning it as the most reliable model for practical deployment in DOI.

Tables 7 and 8 provide a detailed quantitative comparison of various methods used in this study for reconstructing images from simulated and experimental phantom data, focusing on two different datasets denoted as simulated and experimental phantom scenarios.

**Table 7:** Quantitative results on simulation and experimental phantoms ($\mu_{a}$) while DOTNet is used only for simulation cases.

	MSE ($10^{-3}$)		PSNR (dB)		SSIM	
$\mu_{a}$	Simulation	Experimental	Simulation	Experimental	Simulation	Experimental
TR	4.9 ± 2.3	3.3 ± 1.6	23.7 ± 2.4	25.4 ± 2.3	0.54 ± 0.17	0.48 ± 0.1
AUNet	1.1 ± 0.3	1.7 ± 1.7	29.7 ± 1.2	28.5 ±3.0	0.87 ± 0.06	0.68 ± 0.1
Periodic	1.4 ± 1.0	2.0 ± 1.3	29.3 ± 3.1	28.0 ± 3.2	0.86 ± 0.07	0.76 ± 0.1
CAFNet	**0.4 ± 0.1**	**1.4 ± 1.1**	**35.1 ± 3.2**	**30.0 ± 3.8**	**0.90 ± 0.06**	**0.80 ± 0.1**
DOTNet	30 ± 23 (S)		14.9 ± 2.4 (S)		0.65 ± 0.08 (S)	

**Table 8:** Quantitative results on simulation and experimental phantoms for $\mu_{s}^{\prime }$.

	MSE ($10^{-3}$)		PSNR (dB)		SSIM	
$\mu_{s}^{\prime }$	Simulation	Experimental	Simulation	Experimental	Simulation	Experimental
TR	5.1 ± 3.0	2.6 ± 1.1	23.5 ± 2.4	26.2 ± 1.9	0.49 ± 0.14	0.56 ± 0.1
AUNet	1.1 ± 0.3	1.5 ± 0.8	29.7 ± 1.2	28.8 ± 2.2	0.87 ± 0.06	0.66 ± 0.1
Periodic	1.5 ± 1.0	2.0 ± 1.7	29.3 ± 1.5	28.6 ± 3.9	0.85 ± 0.07	0.76 ± 0.1
CAFNet	**0.4 ± 0.1**	**1.2 ± 0.9**	**35.1 ± 3.2**	**30.2 ± 3.1**	**0.90 ± 0.07**	**0.82 ± 0.1**
DOTNet	32 ± 24 (S)		14.95 ± 2.5 (S)		0.66 ± 0.08 (S)	

These tables evaluate the performance of methods including TR, AUNet, Periodic-Net, CAFNet, and DOTNet^36^ (the latter used only for simulation cases) using three key metrics: MSE, PSNR, and SSIM. The results highlight the superior performance of CAFNet across both scenarios.

### 4.3 Resolution Analysis of Reconstructed Images

In this section, the resolution performance of CAFNet compared with TR, Periodic-Net, and AUNet for the cases^14^^,^^16^^,^^31^ illustrated in Figs. 7 and 8 is summarized in Tables 9 and 10. The resolution is evaluated using the CSD metric, which measures the ability to resolve inclusions based on the size and contrast of optical property values relative to the background.^29^^,^^37^ The contrast resolution $R_{\mathrm{cont}.}^{2D}$ is a comparison between the inclusion’s contrast to the surrounding background in the sample

$$R_{0_{\mathrm{cont}.}}^{2D}=\frac{({\overset{¯}{\max}}^{\mathrm{incl}.}/{\overset{¯}{\min}}^{\mathrm{back}.}{)}_{\mathrm{Recon}.}}{({\overset{¯}{\max}}^{\mathrm{incl}.}/{\overset{¯}{\min}}^{\mathrm{back}.}{)}_{\mathrm{Orig}.}},$$ (11)

and,

$$R_{\mathrm{cont}.}^{2D}=\{\begin{array}{ll} 2-R_{0_{\mathrm{cont}.}}^{2D}, & \text{if}R_{\mathrm{cont}.}^{2D}>1\\ R_{0_{\mathrm{cont}.}}^{2D}, & \text{otherwise} \end{array},$$ (12)

where ($\overset{¯}{\max}$) and ($\overset{¯}{\min}$) represent the average of maxima and minima over all of the specified inclusion zones.^29^ The size resolution is designed to evaluate the resolution on the size of all inclusions as

$$R_{\mathrm{size}}^{2D}=\sqrt{\left\{[1-\frac{({\mathrm{RMSE}}^{\mathrm{incl}.}{)}_{\mathrm{Recon}\_\mathrm{to}\_\mathrm{Orig}.}}{({\mathrm{RMSE}}^{\mathrm{incl}.}{)}_{\mathrm{Orig}\_\mathrm{to}\_\mathrm{base}.}}]R_{\mathrm{cont}.}^{2D}\right\}}\equiv \sqrt{R_{0_{\mathrm{size}.}}^{2D}\cdot R_{\mathrm{cont}.}^{2D}}.$$ (13)

**Table 9:** Resolution analysis of simulation case samples.

Case	$R_{\mathrm{cont}.}^{2D}$		$Ro_{\mathrm{size}}^{2D}$					$R_{\mathrm{size}}^{2D}$			$R_{\mathrm{csd}}^{2D}$		
	$\mu_{a}$	$\mu_{s}^{\prime }$	$\mu_{a}$			$\mu_{s}^{\prime }$		$\mu_{a}$	$\mu_{s}^{\prime }$		$\mu_{a}$	$\mu_{s}^{\prime }$	
Tikhonov regularization resolution analysis													
First case	0.70	0.90	0.50			0.51		0.59	0.67		0.65	0.78	
Second case	0.36	0.55	0.28			0.057		0.31	0.12		0.33	0.23	
AUNet resolution analysis													
First case	0.63	0.89	0.56			0.68		0.58	0.78		0.60	0.833	
Second case	0.61	0.77	0.54			0.62		0.57	0.68		0.59	0.72	
Periodic-Net resolution analysis													
First case	**0.82**	0.75	0.34			0.52		0.52	0.62		**0.65**	0.68	
Second case	0.88	0.63	0.61			0.54		0.73	0.57		0.81	0.60	
CAFNet resolution analysis													
First case	0.62	**0.91**		**0.62**	**0.87**		**0.62**			**0.89**	0.62		**0.90**
Second case	**0.94**	**0.83**		**0.72**	**0.68**		**0.82**			**0.75**	**0.88**		**0.79**

Note: bold values indicate the best-performing results among all compared methods.

**Table 10:** Resolution analysis of experimental case samples.

Case	$R_{\mathrm{cont}.}^{2D}$		$Ro_{\mathrm{size}}^{2D}$				$R_{\mathrm{size}}^{2D}$			$R_{\mathrm{csd}}^{2D}$		
	$\mu_{a}$	$\mu_{s}^{\prime }$	$\mu_{a}$		$\mu_{s}^{\prime }$		$\mu_{a}$	$\mu_{s}^{\prime }$		$\mu_{a}$	$\mu_{s}^{\prime }$	
Tikhonov regularization resolution analysis												
First case	0.31	0.27	0.12		0.08		0.19	0.14		0.24	0.20	
Second case	0.58	0.60	0.55		0.55		0.56	0.57		0.57	0.58	
AUNet resolution analysis												
First case	0.47	0.49	**0.13**		0.16		0.25	0.28		0.34	0.37	
Second case	0.79	0.80	**0.65**		0.68		0.71	0.73		0.74	0.76	
Periodic-Net resolution analysis												
First case	0.58	**0.85**	0.10		0.20		0.23	**0.41**		0.36	**0.59**	
Second case	0.67	0.76	0.53		0.59		0.57	0.65		0.61	0.70	
CAFNet resolution analysis												
First case	**0.71**	0.72	**0.13**	**0.22**		**0.31**			0.40	**0.47**		0.54
Second case	**0.81**	**0.81**	0.64	**0.71**		**0.71**			**0.75**	**0.75**		**0.78**

Note: bold values indicate the best-performing results among all compared methods.

The inclusion size resolution was evaluated using the RMSE across the 2D region of interest, quantifying differences between original and reconstructed values. To avoid overestimating inclusion size, contrast resolution was integrated into the metric, providing a unified measure for direct comparison across the three reconstruction methods, i.e., Ref. 37

$$R_{\mathrm{contrast}-\mathrm{size}-\mathrm{detail}}^{2D}=\sqrt{R_{\mathrm{cont}.}^{2D}\cdot R_{\mathrm{size}}^{2D}}.$$ (14)

Higher $R_{\mathrm{csd}}^{2D}$, $R_{\mathrm{size}}^{2D}$, and $R_{\mathrm{cont}.}^{2D}$ indicate closer agreement with ground truth. CAFNet demonstrates superior performance in both simulation and experimental cases, consistently achieving higher resolution metrics than TR, AUNet, and Periodic-Net. These results highlight the stronger capability of CAFNet to reconstruct optical-property images that closely match the ground truth.

### 4.4 Ablation Study

Ablation studies are essential for systematically evaluating the contributions of individual architectural components in neural networks, particularly in tasks requiring high accuracy, such as the reconstruction of optical properties in DOI. The proposed CAFNet architecture is developed to reconstruct $\mu_{a}$ and $\mu_{s}^{\prime }$ images with high fidelity. To assess the role of its key components, namely the FEB, the Fusion block, and the CAB, two structured ablation scenarios are conducted for a test sample having a diameter = 50 mm, freq = 10 MHz, radii of inclusion = 8.56 and 6.15 mm, $\mu_{a}=\;0.0057\;{\mathrm{mm}}^{-1}$, and $\mu_{s}^{\prime }=0.661\;{\mathrm{mm}}^{-1}$.

**Fig. 11:**
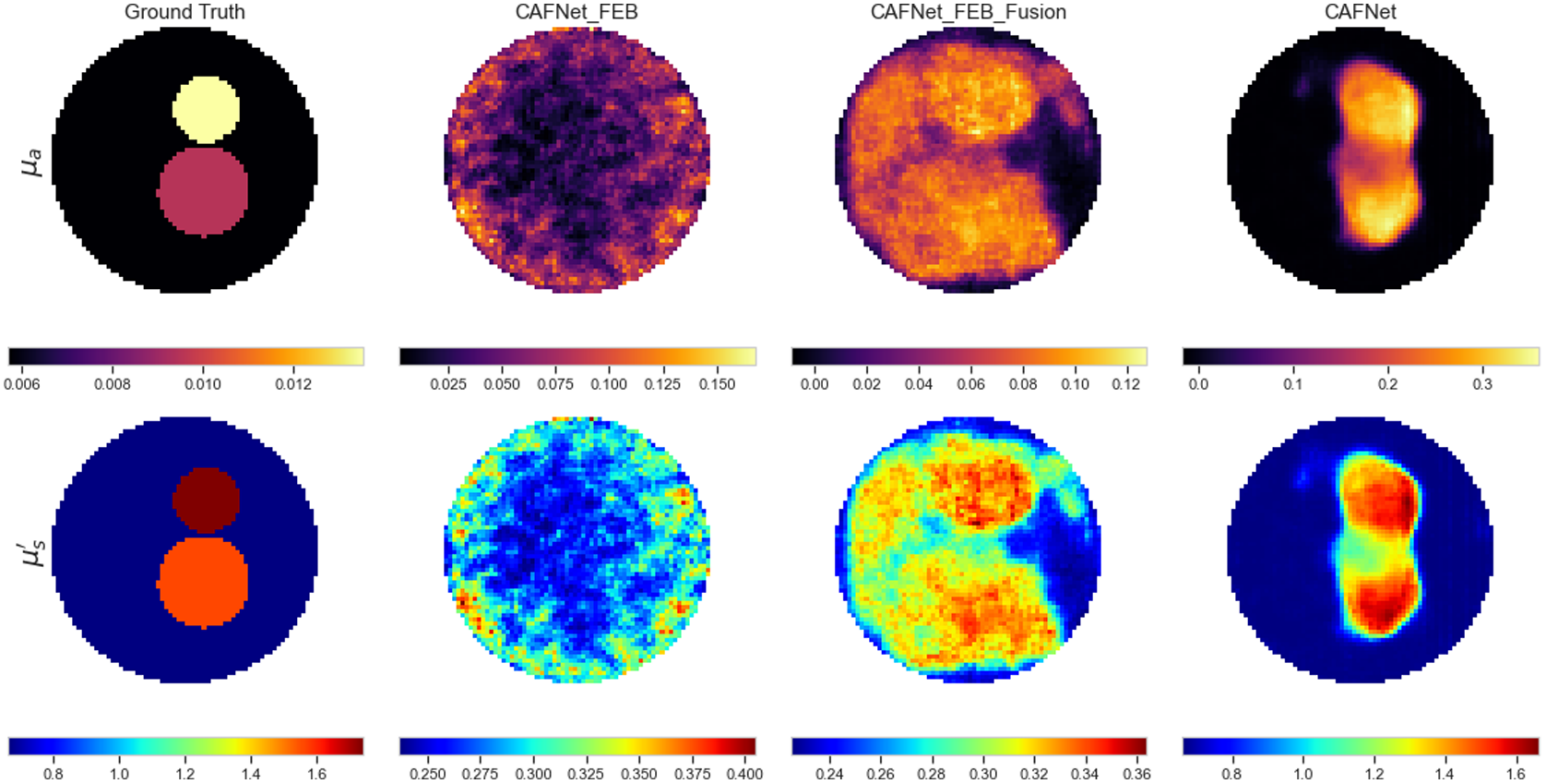
Model evaluation through systematically adding of modules for reconstructing optical property images from left to right: Ground Truth, CAFNet_FEB, CAFNet_FEB_Fusion, and CAFNet.

In the first scenario, a case study was conducted to evaluate the progressive impact of the FEB, fusion block, and CAB on the reconstruction quality. All model configurations were trained for 20 epochs under identical settings to ensure fairness. Quantitative results summarized in Tables 11 and 12 show a steady improvement in performance metrics (MSE, PSNR, and SSIM) for $\mu_{a}$ and $\mu_{s}^{\prime }$ as the modules were gradually incorporated, culminating in the best performance with the complete CAFNet. The reconstructed optical-property maps illustrated in Fig. 11 further exemplify this trend. The model utilizing only the FEB produced noisy and inconsistent reconstructions, indicating limited feature extraction capability. The addition of the fusion blocks improved structural coherence and reduced noise by enhancing feature interaction across channels and spatial dimensions. However, residual artifacts and distortions are still visible, highlighting the need for an additional refinement mechanism. By contrast, the complete CAFNet, integrating the FEB, fusion, and CAB modules, achieved reconstructions with sharp boundaries, enhanced spatial fidelity, and strong agreement with the ground truth. This case study demonstrates that the joint contribution of the fusion and attention mechanisms is essential for achieving optimal reconstruction accuracy and for effectively suppressing noise while preserving fine structural details.

**Table 11:** Performance metrics of CAFNet variants for reconstruction of $\mu_{a}$ under simulation conditions.

$\mu_{a}$	MSE	PSNR (dB)	SSIM
FEB	0.27 ± 0.09	6.03 ± 1.45	0.08 ± 0.01
FEB fusion	0.015 ± 0.01	18.24 ± 2.2	0.48 ± 0.07
CAFNet	**0.0094 ± 0.006**	**21.49 ± 3.28**	**0.75 ± 0.08**

**Table 12:** Performance metrics of CAFNet variants for reconstruction of $\mu_{s}^{\prime }$ under simulation conditions.

$\mu_{s}^{\prime }$	MSE	PSNR (dB)	SSIM
FEB	0.26 ± 0.093	6.0 ± 1.45	0.07± 0.01
FEB fusion	0.014 ± 0.01	18.54 ± 2.34	0.47 ± 0.05
CAFNet	**0.0095 ± 0.006**	**21.33 ± 3.23**	**0.76 ± 0.07**

**Fig. 12:**
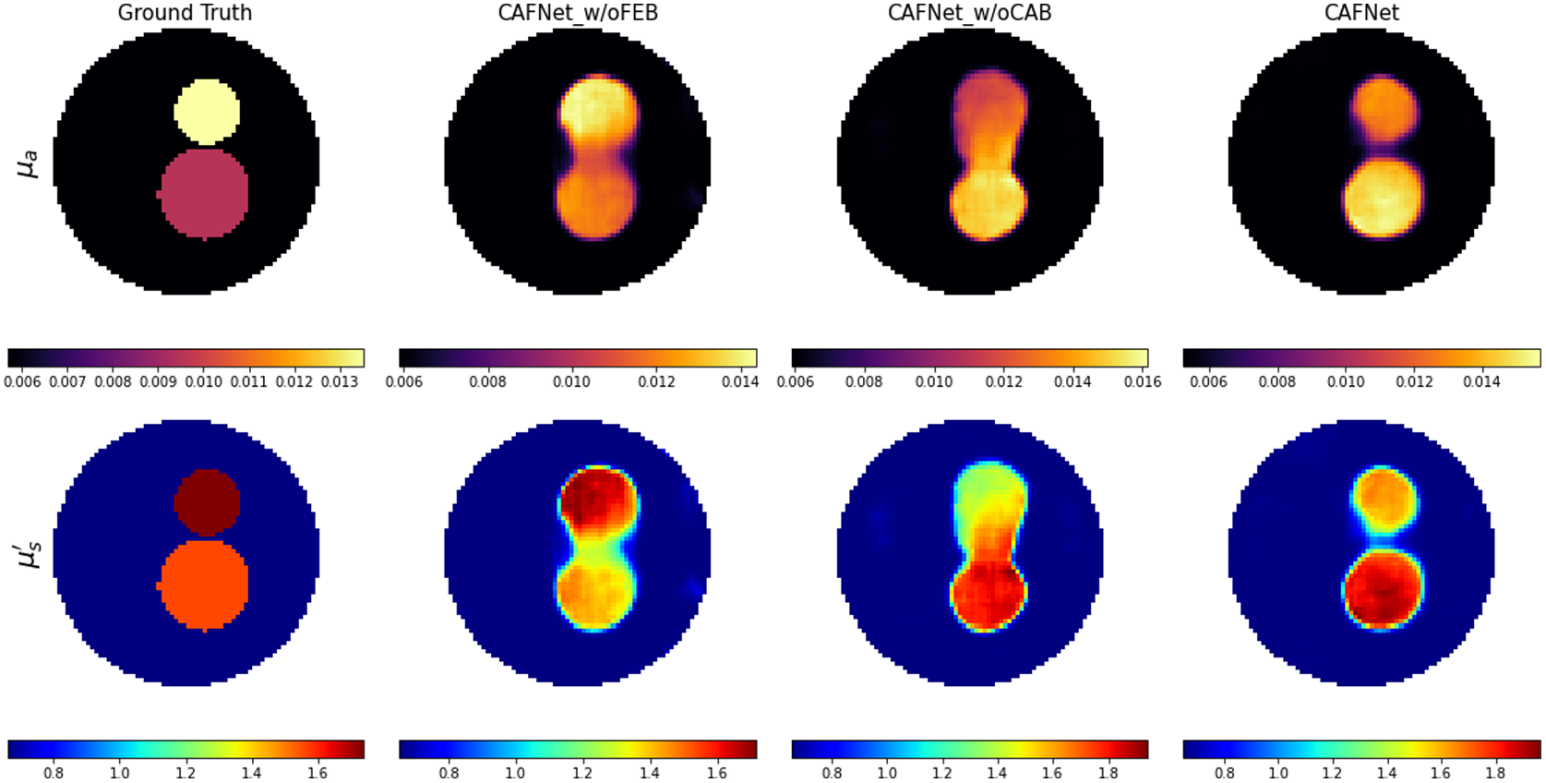
Comparison of optical property reconstruction results for 16×15 synthetic dual inclusion sample from left to right: Ground Truth, CAFNet w/o FEB, CAFNet_ w/o CAB, and CAFNet.

In the second scenario, an ablation study was conducted to evaluate the individual contributions of the CAB and FEB within the CAFNet architecture. The network was trained for 100 epochs under identical conditions across all variants. Quantitative results presented in Tables 13 and 14 reveal a significant decline in performance metrics (MSE, PSNR, and SSIM) when either the CAB or FEB module was removed. The complete CAFNet consistently achieved the best quantitative performance, confirming the complementary roles of both components in enhancing reconstruction accuracy. A qualitative comparison of reconstructed optical-property maps, illustrated in Fig. 12, further supports these findings. The absence of the CAB resulted in blurred reconstructions with substantial loss of structural detail in both the $\mu_{a}$ and $\mu_{s}^{\prime }$ distributions. By contrast, deactivating the FEB while retaining the CAB improved visual quality compared with the “w/o CAB” case but remained inferior to the full CAFNet. These observations indicate that while the FEB contributes to feature representation, the CAB plays a more critical role in maintaining spatial coherence and refining structural details. Overall, the complete CAFNet demonstrates superior reconstruction fidelity with sharp boundaries, accurate localization, and minimal artifacts, underscoring the synergistic impact of the FEB and CAB modules in achieving optimal performance.

**Table 13:** Performance metrics of CAFNet variants for reconstructed $\mu_{a}$ under simulation conditions.

$\mu_{a}$	MSE ($10^{-3}$)	PSNR (dB)	SSIM
CAFNet w/o FEB	1.2 ± 1.1	28.9 ± 2.2	0.85 ± 0.05
CAFNet w/o CAB	1.8 ± 2.2	27.5 ± 1.31	0.83 ± 0.05
CAFNet	**0.4 ± 1.1**	**35.5 ± 3.3**	**0.90 ± 0.06**

**Table 14:** Performance metrics of CAFNet variants for reconstructed $\mu_{s}^{\prime }$ under simulation conditions.

$\mu_{s}^{\prime }$	MSE ($10^{-3}$)	PSNR (dB)	SSIM
CAFNet w/o FEB	1.2 ± 0.9	28.9 ± 2.1	0.85 ± 0.05
CAFNet w/o CAB	1.8 ± 2.2	27.6 ± 1.53	0.84 ± 0.06
CAFNet	**0.4 ± 1.1**	**35.5 ± 3.3**	**0.90 ± 0.07**

**Fig. 13:**
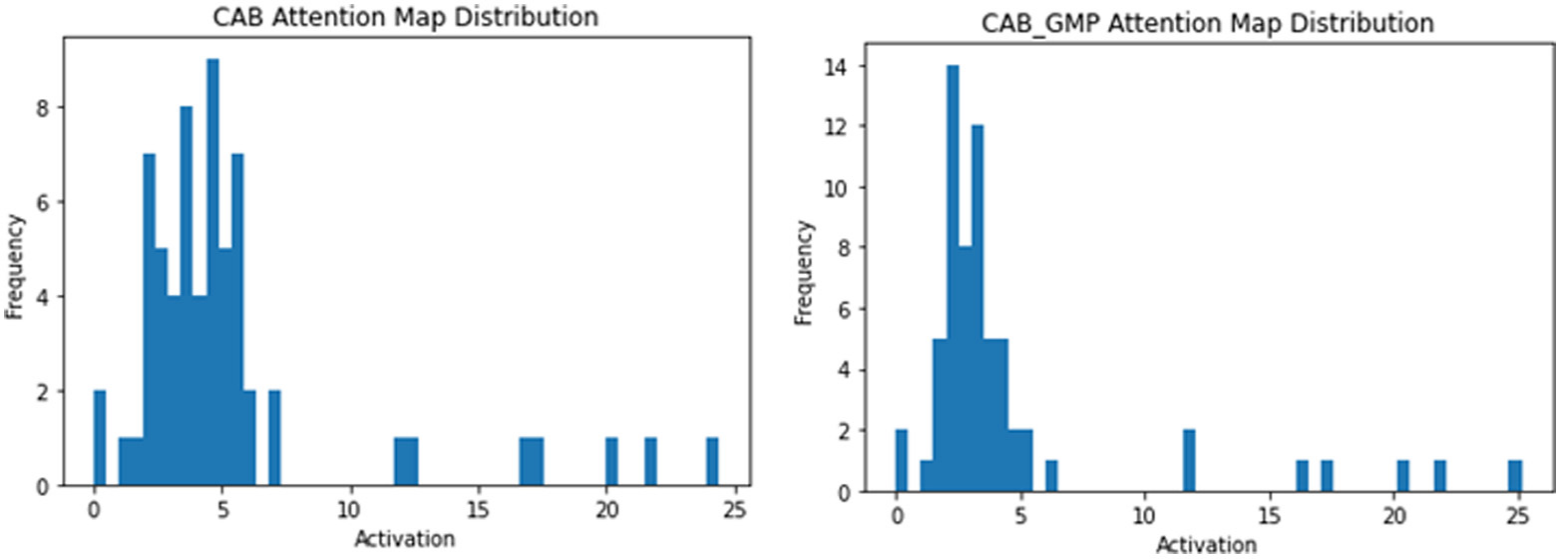
Histogram of spatial attention map activation values, showing the distribution (left) after the CAB and (right) the GMP within it.

The left panel of Fig. 13 shows the distribution of attention weights after initial processing within the CAB, whereas the right panel displays the distribution after the GMP operation, which contributes to the final channel attention map. Both histograms illustrate right-skewed distributions characterized by a majority of low activation values and a sparse presence of significantly higher activation values. This distinctive pattern suggests that the CAB effectively highlights salient features across the channels by selectively assigning higher importance to critical information, thereby guiding the network focus to the most informative channels for subsequent processing.

**Fig. 14:**
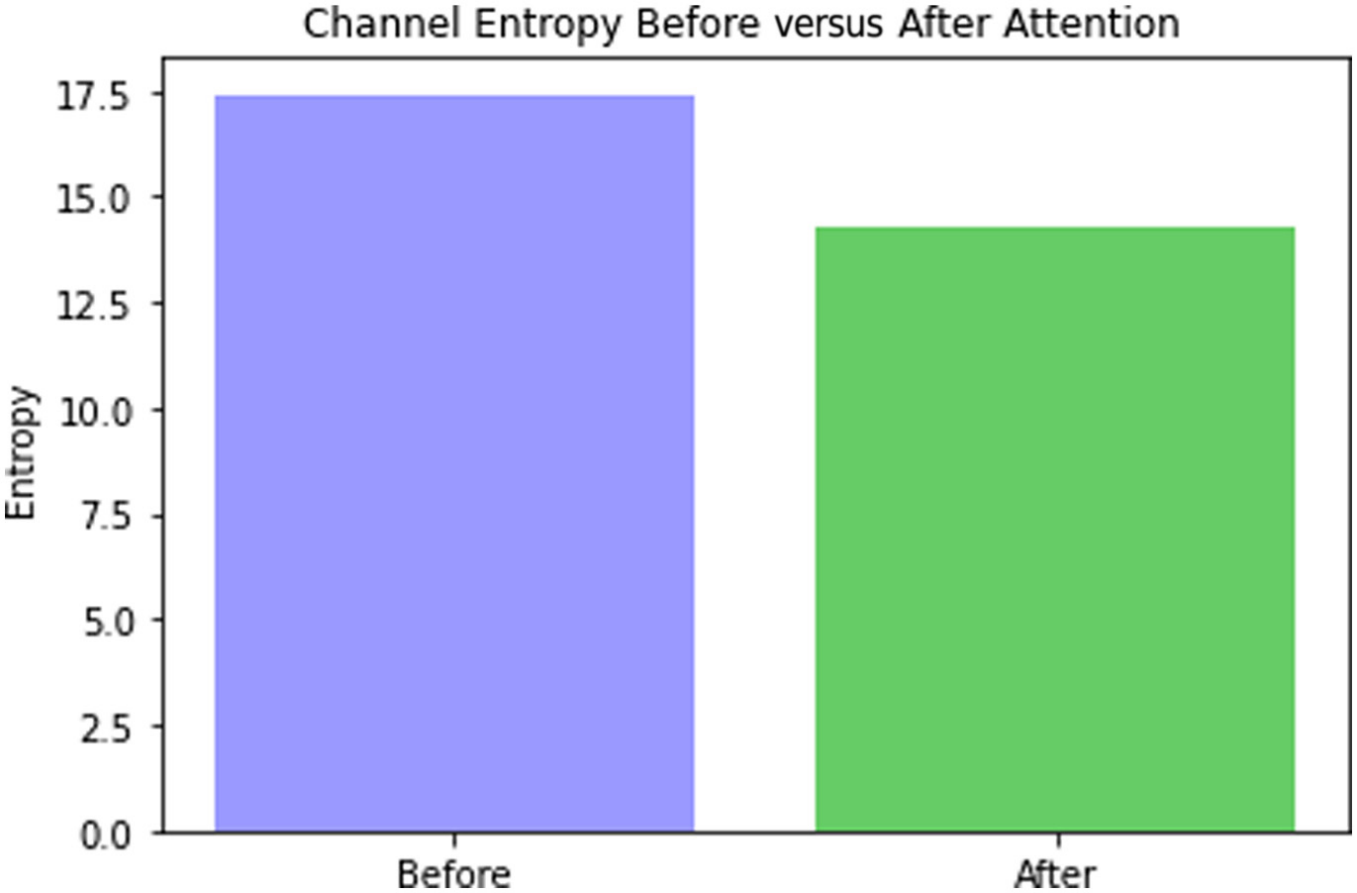
Attention focusing effect: attention entropy (left) 17.5 before and (right) 14.5 after CAB.

Entropy can serve as a measure of uncertainty or diversity in learned representations. Similar to the entropy-based analysis in attention mechanisms by Ref. 38, the proposed study evaluates the change in feature map entropy before and after applying the CAB to quantify information concentration. A noticeable reduction in entropy is observed after the attention mechanism (Fig. 14), indicating that the CAB effectively suppresses redundant information and enhances the discriminative focus of the network. This decrease in channel entropy reflects a selective feature representation, where the model allocates higher importance to the most relevant channels, thereby improving reconstruction consistency.

These ablation results indicate the essential roles of these three modules, especially CAB. The FEB contributes to hierarchical feature representation, the fusion block enables effective multi-scale integration, and CAB enhances channel-wise attention to refine critical regions. Together, these components form a synergistic structure that ensures accurate and robust optical property reconstruction for optical-property images.

## 5 Concluding Remarks

In this study, a novel DNN architecture termed CAFNet is proposed for the accurate reconstruction of optical-property images with a particular emphasis on breast tissue characterization. The CAFNet framework is designed as an end-to-end learning system and has been rigorously evaluated under a variety of test scenarios, including challenging phantom configurations involving multiple inclusions. One of the most demanding test cases considered involves a centrally located inclusion with low optical contrast relative to the surrounding background medium. Such a setup poses significant reconstruction challenges due to weak boundary measurements and signal attenuation, often leading to degraded image quality in traditional methods. Despite these conditions, the proposed CAFNet demonstrates reliable and accurate reconstruction capabilities.

Evaluation of CAFNet is performed using both simulated datasets and experimental phantom data, encompassing a broad spectrum of complexity and realism. Across all test cases, the proposed method consistently yields high-fidelity reconstructions, exhibiting improved spatial localization of inclusions, enhanced delineation of tumor boundaries, and superior preservation of contrast compared with existing baseline approaches. Notably, CAFNet maintains this level of performance even under experimental imaging conditions, which are typically affected by hardware noise, measurement variability, and real-world inconsistencies.

To further assess the effectiveness of key architectural components within CAFNet, an ablation study was conducted focusing on the inclusion of the channel attention mechanism. The CAB is explicitly designed to enhance the representational capacity of the network by adaptively weighting informative feature channels during reconstruction. Removal of the CAB from the CAFNet architecture led to a marked degradation in performance. Optical images reconstructed by the ablated network showed increased noise, blurred tumor boundaries, and diminished agreement with the ground truth contrast profiles. By contrast, the complete CAFNet architecture, incorporating the CAB, delivered significantly sharper reconstructions with reduced artifacts and contrast values that closely matched the original phantom data. These findings substantiate the importance of the CAB in improving the focus of the network on diagnostically relevant features and enhancing its learning capacity in complex imaging scenarios.

Overall, the proposed CAFNet architecture demonstrates consistently strong performance on both synthetic and experimental datasets, confirming its suitability as a practical solution for optical-property image reconstruction. By overcoming key limitations of traditional iterative algorithms and earlier DL models, CAFNet represents a significant advancement in biomedical imaging. Its ability to accurately reconstruct challenging image features, preserve diagnostic details, and generalize across diverse conditions positions. Our future work aims to enhance the proposed DNN by investigating its applicability to non-circular and ring system data, incorporating multiple sources and detectors. We plan to leverage transfer learning to address data distribution shifts and mitigate the impact of random noise, thereby improving the model’s robustness and generalizability.

## 6 Appendix: Experimental Test Dataset

Table 15 provides the complete experimental test dataset employed in the study, along with the associated parameters previously summarized in Table 2.

**Table 15:** Employed experimental test dataset and associated parameters.

	d (mm)	f (MHZ)	r (mm)	$L_{-\mathrm{OC}}$ (mm)	θ (deg)	$\mu_{a}$ (${\mathrm{mm}}^{-1}$)	$\mu_{s}^{\prime }$ (${\mathrm{mm}}^{-1}$)	C_a	C_sp
1	80	20	10	25	180	0.006	0.6	4	4
2	50	20	5	15	180	0.006	0.6	3	3
3	50	20	5	15	180	0.006	0.6	4	4
4	50	20	10	12.5	180	0.006	0.6	4	4
5	50	20	5, 5	12.5, 12.5	225, 135	0.006	0.6	4, 4	4, 4
6	50	20	5	10	270	0.0079	0.6	4	4
7	50	20	5	10	225	0.0079	0.6	4	4
8	50	20	5	10	180	0.006	0.6	4	4
9	50	20	5.5	10	180	0.006	0.6	4	4
10	50	20	5.5	12.5	180	0.0079	0.6	4	4
11	50	20	5	12.5	180	0.0079	0.6	4	4
12	50	20	5, 5	12.5, 12.5	90, 270	0.0079	0.6	4, 4	4, 4
13	80	20	5	20	180	0.0074	0.85	3	0.89
14	50	20	5, 5	12.5, 12.5	135, 225	0.0079	0.6	4, 4	4, 4
15	80	20	10	25	270	0.006	0.6	4	4
16	50	20	5, 5	15, 15	240, 120	0.0079	0.6	3, 2	3, 2
17	80	70	5, 5	25, 25	225, 135	0.008	0.3	3, 3	3, 3
18	80	50	5, 5	25, 25	225, 135	0.008	0.3	3, 3	3, 3
19	80	20	5, 5	25, 25	225, 135	0.008	0.3	3, 3	3, 3
20	50	20	5	10	180	0.006	0.6	3	3
21	50	20	5, 5	15, 15	180, 90	0.006	0.6	2, 2	2, 2
22	80	20	7.5	25	180	0.01	1	4	4

N.B.: L__OC_ (off-center distance); C_a (inclusion to background contrast of $\mu_{a}$); C_sp (inclusion to background contrast of $\mu_{s}^{\prime }$).

## Data Availability

The code and sample dataset are publicly available on GitHub at [https://github.com/SDMPLab/CAFNet]; the full dataset is available upon reasonable request.
